# The China plant trait database version 2

**DOI:** 10.1038/s41597-022-01884-4

**Published:** 2022-12-15

**Authors:** Han Wang, Sandy P. Harrison, Meng Li, I. Colin Prentice, Shengchao Qiao, Runxi Wang, Huiying Xu, Giulia Mengoli, Yunke Peng, Yanzheng Yang

**Affiliations:** 1 Department of Earth System Science, Ministry of Education Key Laboratory for Earth System Modeling, Institute for Global Change Studies, Tsinghua University, Beijing, 100084 China; 2 School of Archaeology, Geography and Environmental Sciences (SAGES), University of Reading, Reading, RG6 6AH United Kingdom; 3 Co-Innovation Center for Sustainable Forestry in Southern China, College of Biology and the Environment, Nanjing Forestry University, Nanjing, 210037 China; 4 Georgina Mace Centre for the Living Planet, Department of Life Sciences, Imperial College London, Silwood Park Campus, Buckhurst Road, Ascot, SL5 7PY United Kingdom; 5 School of Biological Sciences, University of Hong Kong, Pok Fu Lam Road, Hong Kong SAR, China; 6 Department of Environmental Systems Science, ETH, Universitätsstrasse 2, 8092 Zurich, Switzerland; 7 Swiss Federal Institute for Forest, Snow and Landscape Research WSL, Zürcherstrasse 111, 8903 Birmensdorf, Switzerland; 8 State Key Laboratory of Urban and Regional Ecology, Research Center for Eco-environmental Sciences, Chinese Academy of Sciences, Beijing, 100085 China

**Keywords:** Biodiversity, Biogeography, Ecophysiology, Ecosystem ecology, Carbon cycle

## Abstract

Plant functional traits represent adaptive strategies to the environment, linked to biophysical and biogeochemical processes and ecosystem functioning. Compilations of trait data facilitate research in multiple fields from plant ecology through to land-surface modelling. Here we present version 2 of the China Plant Trait Database, which contains information on morphometric, physical, chemical, photosynthetic and hydraulic traits from 1529 unique species in 140 sites spanning a diversity of vegetation types. Version 2 has five improvements compared to the previous version: (1) new data from a 4-km elevation transect on the edge of Tibetan Plateau, including alpine vegetation types not sampled previously; (2) inclusion of traits related to hydraulic processes, including specific sapwood conductance, the area ratio of sapwood to leaf, wood density and turgor loss point; (3) inclusion of information on soil properties to complement the existing data on climate and vegetation (4) assessments and flagging the reliability of individual trait measurements; and (5) inclusion of standardized templates for systematical field sampling and measurements.

## Abstract (summary)

**Table:** 

Measurement(s)	plant functional traits
Technology Type(s)	plant functional traits
Sample Characteristic - Organism	Plants
Sample Characteristic - Location	China

## Background & Summary

Plant functional traits are observable characteristics that reflect eco-evolutionary responses to environmental conditions^1–4^. Plant traits have been used to investigate the responses of vegetation to environmental conditions at scales from individuals to biomes^5–8^. There is a wealth of empirical and theoretical analyses of the relationships between specific traits, or groups of traits, in relation to specific environmental constraints, including climate, nutrient availability and disturbance^9–13^. The creation of regional and global trait data sets in recent decades^14–18^ has stimulated research spanning community and functional ecology, biodiversity conservation, ecosystem and landscape management, biogeography and land-surface modelling^19–24^.

The first version of the China Plant Trait Database (CPTDv1) includes information on a wide range of morphometric, physical, chemical and photosynthesis traits and provides a sampling of the different types of vegetation in China^18^. The sampling sites represent a wide range of environmental conditions: growing season temperatures, as measured by the accumulated temperature sum above 0 °C (GDD0), range from close to zero to over 9000 °C days; aridity, as measured by the ratio of actual to equilibrium evapotranspiration (α) ranges from hyper-arid to saturated. Most global major biomes are represented in China, with the exception of Mediterranean-type vegetation. The CPTDv1 has been used to address fundamental questions such as the relative importance of species replacement versus phenotypic plasticity in determining observed trait-environment relationships^25–27^, the dimensionality of leaf functional traits^28^, the predictability of plant biochemical and structural traits^29,30^, the relationship of morphological traits to climate gradients^31^, and plant eco-physiological responses to climate change^28,32^.

**Fig. 1:**
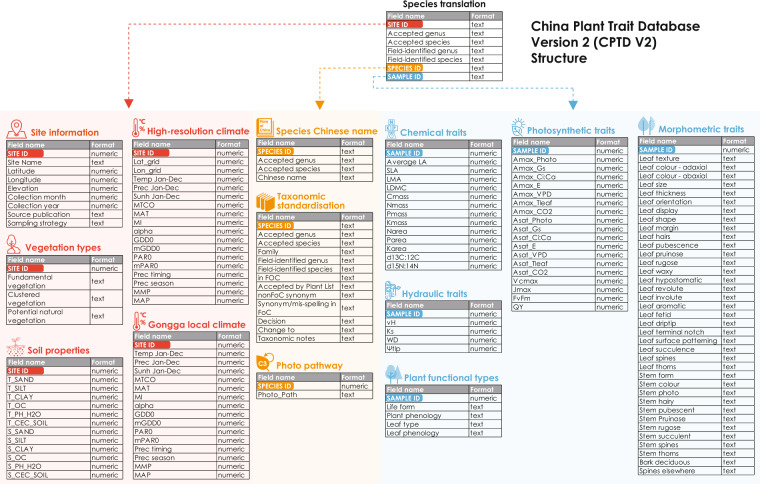
The structure of the China Plant Trait Database Version 2. Each block represents one table in the database. The tables providing the information on sites (in red), species (in yellow), and samples (in blue) can be linked via ‘SITE ID’, ‘SPECIES ID’ and ‘SAMPLE ID’. The table called species translation is the central table linking the three identifying keys. The definition and detailed information of all variables are provided in Tables 1-10 and Supplementary Tables 1-4.

**Fig. 2:**
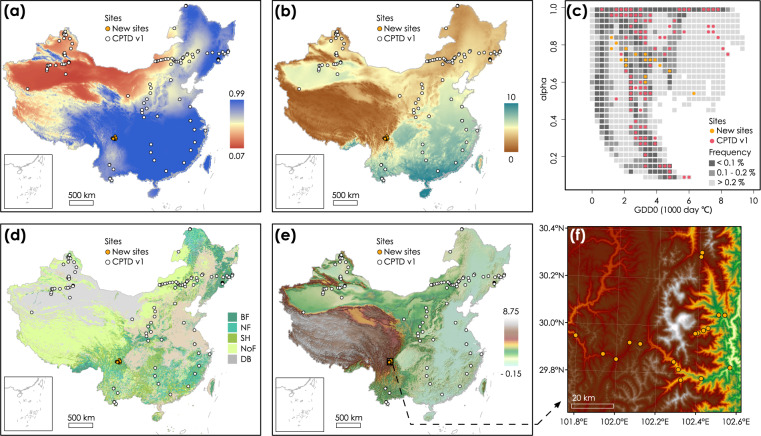
Location of sites in the China Plant Trait Database version 2. The site locations are imposed on the maps of (a) the Presley- Taylor coefficient as the moisture index (α, unitless), (b) the growing degree days (GDD0, in 1000 ˚C day), (d) biome types (BF: Broadleaf Forest, NF: Needleleaf Forest, SH: Shrubland, NoF: NonForest, DB: Desert and Bare ground) and (e) elevation (km). (c) The site locations are projected onto the climate space defined by α and GDD0. The grey cells show the frequency distribution of 10km grid cells across the whole of China in this climate space. (f) the zoom-in locations of new sites along the elevation gradient in Gongga Mountain.

Nevertheless, there are important gaps in the CPTDv1. The site coverage is biased towards tropical and temperate/boreal climates. Alpine environments are poorly represented, although about 8% of the world’s land surface is above 1500 m altitude^33^ and these regions are suffering faster rates of climatic change than lowland areas^34^. Furthermore, some important functional traits mediating plant eco-physiological processes, such as plant hydraulics and biomass allocation^35,36^, are poorly represented in the CPTDv1. To overcome these deficiencies in the existing database, we have created an updated version of the CPTD (CPTDv2). This uses the same basic structure as the previous version, but with additional fields and tables to accommodate new data types. The CPTDv2 is provided by 14 tables in the format of csv and xlsx. The different tables of information on site, species or samples are linked via three key identifiers of ‘SiteID’, ‘SpeciesID’ and ‘SampleID’. The table ‘Species_translations_v2.csv’ serves as the the central table achieving the link among those identifying keys (Fig. 1, Tables 1–10, Supplementary Tables 1–4). The database now includes data from 18 new sites in the Gongga Mountains on the eastern edge of the Tibetan Plateau to improve the coverage of alpine vegetation (Fig. 2). Two elevation transects ranging from 1143 m to 4361 m were sampled from both wet and dry environments in parallel. The database also comprises measurements of hydraulic traits, specifically wood density, specific sapwood conductivity, the sapwood to leaf area ratio (Huber value) and turgor loss point. Hydraulic traits were measured together with other photosynthetic and leaf biochemistry traits, allowing systemically analysis of the co-ordination or trade-offs among those traits at a plant and community level^13^. Although the CPTDv1 provided high-resolution information on climate and vegetation, this has now been further improved. In addition, information on soil properties have been extracted for all the sites to facilitate analyses of soil effects on plant traits. The new version of the database contains 2949 samples from 1529 species across 140 field sites in total (Fig. 2).

**Table 1:** Species translations.

Field name	Definition	Number of records
Site ID	unique identifier for each site	2949
Original genus	genus name recorded in the field	2926
Original species	species name recorded in the field	2926
Accepted genus	genus name accepted with the species standardization protocol	2926
Accepted species	species name accepted with the species standardization protocol	2926
Species ID	unique identifier for each species	2949
Sample ID	unique identifier for each sample	2949

**Table 2:** Sites.

Field name	Definition	Units/ coding
Site ID	unique identifier for each site	NA
Site Name	site name as given by original authors or as defined by us where there was no unique name given to the site	NA
Latitude	latitude	decimal degrees
Longitude	longitude	decimal degrees
Elevation	above sea level	meters
Collection month	month of sampling, and the number represents the calendar month	NA
Collection year	year of sampling	NA
Source	publications from which the observations were obtained	NA
Sampling strategy	how species were sampled in field	A: sampling of a limited number of key species at a site;
		D: sampling of dominant species only;
		SS: stratified sampling;
		PSS: sampling of a limited number of strata

**Table 3:** Taxonomic standardization.

Field name	Definition	Number of records
Original genus	genus name recorded in the field	1568
Original Species	species name recorded in the field	1568
in FOC	whether or not the original species name recorded in the Flora of China	NO (242); YES (1314); NA (15); Mis-spelling (12)
Accepted by Plant List	whether or not the original species name accepted by the Plant List	NO (249); YES (1296); NA (15); Mis-spelling (2); unsolved (21)
nonFoC synonym	whether or not the original species recorded as synonym in multiple sources of the Plant list, Plants of the World Online, Tropicos and the Virtual Herbarium of China	NO (15); YES (233)
Synonym/mis-spelling in FoC	whether or not the original species given as synonym or mis-spelt in the Flora of China	NO (123); YES (163); mis-spelling (1)
Decision	change or keep the original species name recorded in the field	CHANGE (180); KEEP (1392); Keep by default (11)
Change to	species name after taxonomic standardisation when changes occur	172
Taxonomic notes	notes on the taxonomic standardisation when changes occur	181
Accepted genus	genus name accepted with the species standardization protocol	1568
Accepted species	species name accepted with the species standardization protocol	1568
Family	family name of the accepted species	1570
Species ID	unique identifier for each species	1583

**Table 4:** Chinese names.

Field name	Definition	Number of records
Species ID	unique identifier for each species	1420
Accepted genus	genus name accepted with the species standardization protocol	1420
Accepted species	species name accepted with the species standardization protocol	1420
Chinese name	Chinese name of the species	1420

**Table 5:** Photosynthesis pathway.

Field name	Definition	Number of records
Species ID	unique identifier for each species	1243
Photo pathway	the photosynthetic pathway of each species	C3 (1194), C4 (47) and CAM (2)

**Table 6:** Physical and chemical traits.

Field name	Definition	Units	Number of observations	Min	Median	Max
Sample ID	unique identifier for each sample	NA	2888			
LA	leaf area	m^2^	2408	0.00000017	0.0016	0.24
SLA	specific leaf area	m^2^/kg	2544	1.64	16.58	83.38
LMA	leaf mass per area	kg/m^2^	2544	0.012	0.060	0.61
LDMC	leaf dry matter content	mg/g	2003	62.09	338.94	1000
C_mass_	leaf carbon content	g/kg	1817	251.241	453.01	693.50
N_mass_	leaf nitrogen content	g/kg	2315	3.41	18.79	60.00
P_mass_	leaf phosphorus content	g/kg	1689	0.11	2.08	7.87
K_mass_	leaf potassium content	g/kg	1122	0.12	12.25	84.33
N_area_	leaf nitrogen content per unit area	g/m^2^	2305	0.11	1.16	8.77
P_area_	leaf phosphorus content per unit area	g/m^2^	1685	0.00024	0.12	0.95
K_area_	leaf potassium content per unit area	g/m^2^	1119	0.0030	0.64	7.22
d^13^C:^12^ C	the ratio of ^13^C to ^12^C stable isotopes in the leaf	unitless	1413	−39.07	−29.40	−11.83
d^15^N:^14^ N	the ratio of ^15^N to ^14^N stable isotopes in the leaf	unitless	1152	−7.40	−1.54	12.14
flagged	traits with some potential problems or unrealistic values for each sample

**Table 7:** Hydraulic traits.

Field name	Definition	Units	Number of observations	Min	Median	Max
Sample id	unique identifier for each sample	NA	427			
v_H_	the ratio of sapwood to leaf area	m^2^/m^2^	292	0.000021	0.00015	0.00171
K_S_	sapwood-specific hydraulic conductivity	kg/s/m/MPa	186	0.032	0.83	4.95
WD	wood density	g/cm^3^	186	0.27	0.57	3.10
Ψ_tlp_	leaf water potential at turgor loss point	MPa	181	−2.98	−1.82	−0.65

**Table 8:** Plant Functional Types.

Field name	Definition	Category	Number of observations
Sample ID	unique identifier for each sample	NA	2949
Life form	assignment to life form	tree	2947
		small tree	
		low to high shrub	
		erect dwarf shrub	
		prostrate dwarf shrub	
		trailing shrub	
		liana	
		climber	
		forb	
		cushion forb	
		rosette forb	
		graminoid	
		bamboo	
		cycad	
		geophyte	
		stem succulent	
		succulent	
		pteridophyte	
		epiphyte	
		parasite	
Plant phenology	description of longevity of the plant itself	perennial	2931
		biennial	
		annual	
Leaf type	description of leaf type	aphyllous	2932
		broad	
		needle	
		scale	
Leaf phenology	assignment based on longevity of leaves for woody plants	deciduous	1833
		semi-deciduous	
		leaf-exchanger

**Table 9:** Vegetation.

Field name	Definition	Number of vegetation classes
Site ID	unique identifier for each site	NA
Fundamental vegetation type	the fundamental vegetation type extracted from Vegetation Map of China	34
Clustered vegetation	the k-means clustered vegetation type	9
Potential natural vegetation	the vegetation type extracted from the global biome map	6

**Table 10:** Soil.

Field name	Definition	Units	Number of observations
Site ID	unique identifier for each site	NA	140
T_SAND	topsoil sand fraction	%	140
T_SILT	topsoil silt fraction	%	140
T_CLAY	topsoil clay fraction	%	140
T_OC	topsoil organic carbon	%	140
T_PH_H2O	topsoil pH (H2O)	Unitless	140
T_CEC_SOIL	topsoil cation exchange capacity	cmol/kg	140
S_SAND	subsoil sand fraction	%	132
S_SILT	subsoil silt fraction	%	132
S_CLAY	subsoil clay fraction	%	132
S_OC	subsoil organic carbon	%	132
S_PH_H2O	subsoil pH (H2O)	Unitless	132
S_CEC_SOIL	subsoil cation exchange capacity	cmol/kg	132

Analyses made using the CPTDv1 have identified some potential problems or unrealistic values for individual data points. In CPTDv2, outliers and other problematic measurements have been systematically identified and flagged. Finally, it is widely recognised that differences in field protocols applied can affect reported trait measurements and introduce uncertainties in analyses of these data^37,38^. Since the intention is to continue to expand the CPTD and include data from multiple groups working in China, we also document the measurement protocols and provide templates for field sampling and trait measurements.

## Methods

### Site selection and sampling strategy

Field sites (Table 1) were selected to represent typical natural vegetation types showing little or no signs of disturbance. Although much of the natural vegetation of China has been altered by human activities, there are still extensive areas of natural vegetation. Access to these areas is facilitated by the existence of a number of ecological transects^39,40^, the ChinaFlux network (http://www.chinaflux.org) and the Chinese Ecosystem Research Network (http://www.cern.ac.cn/0index/index.asp).

About half the sites in CPTDv1 used a stratified sampling approach and this approach was used at all of the new sites added in the CPTDv2. This sampling strategy involves sampling the dominant species within each vegetation stratum so as to be able to characterise trait values at community level^18^. Specifically, a total of 25 trees, 5 shrubs, 5 lianas or vines, and 5 understorey species (grasses, forbs) were sampled at each site. When there were less than 25 trees at a site, all of the tree species were sampled and additional examples from the other categories were included up to the maximum of 40 species. If there are more than the maximum sampling number in any one category, then the dominant (i.e. most common) representatives of each category were sampled. Sampled individuals of each species were mature, healthy plants. In principle, sun leaves (i.e. leaves in the canopy and fully exposed to sunlight) were sampled. For true shade-tolerant and understory species, the sampled individuals were those in well-lit environments and isolated to minimize interactions with other individuals.

Nineteen sites from Xinjiang included in CPTDv1 used a simplified sampling strategy, where only canopy species were sampled. Sixteen sites from Xinjiang were particularly depauperate and thus only a limited number of species were sampled without consideration of abundance. These sites are retained in the database because they sample extremely arid location with α typically less than 0.25

### Species identification and taxonomic standardisation

**Fig. 3:**
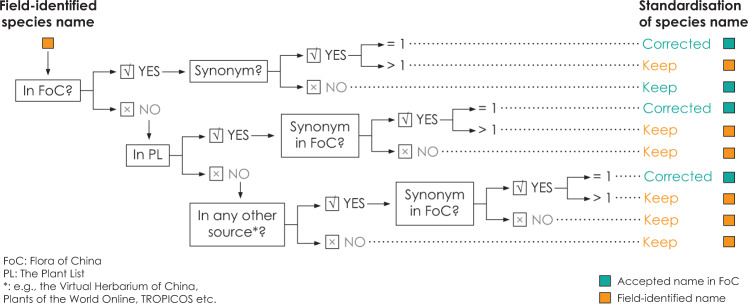
Flowchart showing the decision tree used to determine the names used in the China Plant Database (accepted names) and encapsulated in the Taxonomic Standardization table. ‘=1’ and ‘>1’ indicate the number of Synonyms is equal or more than one.

Sampled plants were identified in the field by a taxonomist familiar with the local vegetation, most usually using a regional flora. Species names were subsequently standardised using the online version of the Flora of China (http://www.efloras.org/flora_page.aspx?flora_id=2). Where field-identified species were not accepted or included in the Flora of China, and thus could not be assigned unambiguously to an accepted taxonomic name, we cross-checked whether the species were listed in the Plant List (http://www.theplantlist.org/) (or alternative sources such as the Virtual Herbarium of China, Plants of the World Online or TROPICOS) in order to identify synonyms for these accepted names that were recognised by the Flora of China. In cases where we were unable to identify an accepted name consistent with the Flora of China, we retained the field-assigned name by default (Fig. 3). The decisions about taxonomy are described in the CPTDv2 table “Taxonomic Standardisation” (Table 2). The names assigned originally in the field and the accepted standardized names used in the database are given in the CPTDv2 table “Species Translations” (Table 3). When species were recognised in the Flora of China, we provide the Chinese translation of the species name. The written Chinese nomenclature system does not follow the Linnaean system, so this table of “Species Chinese Name” is designed to facilitate the use of the database by botanists in China (Table 4). There are no translations of names that are not recognized by the Flora of China and are used in the database by default.

### Dataset collection methods

#### Photosynthetic pathway

Information on photosynthetic pathway (Table 5) was obtained for each species from the literature. There are a large number of literature compilations on the photosynthetic pathway of Chinese plants (e.g.^41–46^. Where this information was not available from Chinese studies we used similar compilations from other regions of the world (e.g.^47–52^. Since C_4_ plants have much less carbon discrimination than C_3_ plants, the measurements on δ^13^C were also used as an indicator of the photosynthetic pathway^53–56^. δ^13^C value of –20‰ was applied as a threshold of C_3_ photosynthetic pathway distinction^54^. Information about photosynthetic pathway was not included for a species unless confirmed from the literature or δ^13^C measurements.

#### Leaf physical and chemical traits

Physical and chemical properties (Table 6) were measured on samples collected in the field following standard methods^37^. At least 10 g of leaves were collected for each species. Sunlit leaves of tree species were obtained with long-handled twig shears. The samples were subdivided for the measurement of specific leaf area, leaf dry matter content and the contents of carbon, nitrogen, phosphorus and potassium. Recorded values were the average of three replicates. Leaf area was determined by scanning five leaves (or more in the case of small leaves, to make up a total area ≥20 cm^2^ per species) with a laser scanner. Areas (Average LA) were measured using Photoshop on the scanned images. Leaf fresh weight was measured in the field. Dry weight was obtained after air drying for several days and then oven drying at 75 °C for 48 hours. Leaf dry matter content (LDMC) was expressed as leaf oven-dry weight divided by fresh weight. Specific leaf area (SLA) was then expressed as the ratio between leaf area and leaf dry mass. LMA is the inverse of SLA. Leaf carbon content (C_mass_) was measured by the potassium dichromate volumetric method and leaf nitrogen content (N_mass_) by the Micro-Kjeldahl method. Leaf phosphorus (P_mass_) was analysed colorimetrically (Shimadzu UV-2550). Leaf potassium (K_mass_) was measured by Flame Atomic Emission Spectrophotometry (PE 5100 PC). The area-based leaf chemical contents (C_area_, N_area_, P_area_, K_area_) were derived as a product of mass-based content and LMA. δ^13^C (d^13^C:^12^C) and δ^15^N (d^15^N:^14^N) were measured using the Isotope Ratio Mass Spectrometer (Thermo Fisher Scientific Inc., USA; Finnigan Corporation, San Jose, CA).

#### Photosynthetic traits

Several different methods were used to characterise photosynthetic traits (Supplementary Table 1). Chlorophyll fluorescence measurements were made at the sites along Northeast China Transect. These measurements were recorded as the potential (Fv/Fm) and actual (QY) rates of photosynthetic electron transport. QY is correlated with photosynthetic rate, although it also includes the diversion of electrons to non-photosynthetic activities such as the elimination of reactive oxygen species^57^. Measurements of photosynthetic traits at most of the sites (about 68% of samples with photosynthetic measurements) were derived from leaf gas-exchange measurements in light-saturated conditions under either ambient or high CO_2_ levels, made with a portable infrared gas analyser (IRGA) system (LI-6400; Li-Cor Inc., Lincoln, NB, USA). Sunlit terminal branches from the upper canopy were collected and re-cut under water immediately prior to measurement. Measurements were made in the field with relative humidity and chamber block temperature close to that of the ambient air at the time of measurement, and a constant airflow rate (500 μmol s^−1^). The maximum capacity of carboxylation (*V*_cmax_) and electron-transport (*J*_max_) were calculated from the light-saturated rate of net CO_2_ fixation at ambient and high CO_2_ level respectively using the one-point method for *V*_cmax_^58^ and two-point method for *J*_max_^59^. Although it was indicated that applying one-point method could result in around 20% error in measuring photosynthetic capacity^60^, this time-saving method indeed allows much more samples to be measured in the field. For sites in CPTDv1, the *V*_cmax_ and *J*_max_ values were made on a single specimen of each species at each site, due to the time-consuming nature of the measurement. For the newly collected sites in CPTDv2, for each species the *V*_cmax_ and *J*_max_ were measured on three samples collected from three individual tress. The average values were recorded in the database. For *V*_cmax_ measurements, the CO_2_ level was set as the ambient atmospheric CO_2_ level, ranging from 380 ppm to 400 ppm. The leaves were exposed to a typical photosynthetic photon flux density (PPFD) of 1800 μmol m^−2^ s^−1^ with the light source. Pre-processing method was applied to determine the saturating PPFD for alpine plants, which goes up to 2000 μmol m^−2^ s^−1^ in the high elevation sites from Mountain Gonga. For *J*_max_ measurements, the CO_2_ level was set as 1500 ppm or 2000 ppm to avoid any limitation on photosynthesis via carboxylation.

There are a few cases (1 site from Cai, *et al*.^61^, and 8 sites from Zheng and Shangguan^62^, Zheng and Shangguan^63^), where field-measured ratio of leaf internal- to ambient-CO_2_ concentration (*c*_i_:*c*_a_) were not provided. In these cases, estimates of the *c*_i_:*c*_a_ ratio were made from δ^13^C measurements using the method of^64^ to calculate isotopic discrimination (Δ) from δ^13^C (correcting for atmospheric δ^13^C, approximated as a function of time of collection and latitude), and the Ubierna and Farquhar^65^ method to calculate isotopic discrimination (Δ) from δ^13^C considering discrimination during stomatal diffusion and carboxylation. The R code for calculating *V*_cmax_ and *J*_cmax_ from original data was provided (seeing Code availability).

#### Hydraulic traits

CPTDv2 contains information on four important hydraulic traits: specific sapwood conductivity, the sapwood to leaf area ratio (Huber value, v_H_), turgor loss point and wood density (Table 7). Hydraulic traits were measured on branches with a diameter wider than 7 mm, cut as close to the bifurcation point as possible to minimize any effect of measurement location on measured area. A section was taken from the part of the branch nearest to the bifurcation point, and the cross-sectional area of the xylem was measured at both ends of this section using digital calipers. Sapwood area was calculated as the average of these two measurements. All leaves attached to the branch were removed and dried at 70 °C for 72 hours before weighing. The total leaf area was obtained from dry mass and LMA. v_H_ was calculated as the ratio of sapwood area and leaf area. The v_H_ value recorded for each species at each site was the average of three measurements made on branches from different individuals.

Five branches from at least three mature individuals of each species at each site were collected, wrapped in moist towels and sealed in black plastic bags, and then immediately transported to the laboratory. All the samples were re-cut under water, put into water and sealed in black plastic bags to rehydrate overnight. Sapwood-specific hydraulic conductivity, (*K*_S_) was measured using the method of Sperry, *et al*.^66^. Segments (10–15 cm length) were cut from the rehydrated branches and flushed using 20 mmol L^−1^ KCl solution for at least 30 minutes (to remove air from the vessels) until constant fluid dripped from the section. The segments were then placed under 0.005 MPa pressure to record the time (*t*) they took to transport a known water volume (*W*, m^3^). Length (*L*, m), sapwood area of both ends (*S*_1_ and *S*_2_, m^2^) and temperature (*T*_m_, °C) were recorded. Sapwood-specific hydraulic conductivity at measurement temperature (*K*_S,m_, mol m^−1^ s^−1^ MPa^−1^) was calculated using Eq. (1). This was transformed to *K*_S_ at mean maximum temperature during the growing season (*K*_S,gt_) and standard temperature (*K*_S25_) following Eqs. (2–3):

1 $${K}_{S,m}=\{W\,L{\rho }_{w}/[0.005\,t({S}_{1}+{S}_{2})/2]\}(1000/\,18)$$

2 $${K}_{S,t}={K}_{S,m}{\eta }_{m}/{\eta }_{t}$$

3 $$\eta =1{0}^{-3}exp[A+B/\,(C+T)]$$

where η_m_ and η_t_ (Pa s) are the water viscosity at measurement temperature and transformed temperature (i.e. mean maximum daytime temperature during the growing season and at a standard temperature of 25 °C), respectively, and ρ_w_ (kg m^−3^) is the density of water. The parameter values used in Eq. (3) were *A* = −3.719, *B* = 580 and *C* = −138^67^.

A small part of each sapwood segment was used to measure wood density, the ratio of dry weight to volume of sapwood. After removal of bark and heartwood, the volume of sapwood was measured by displacement and the sapwood dry weight was obtained after drying at 70 °C for 72 hours to constant weight.

The method described by Bartlett, *et al*.^68^ was used for the rapid determination of turgor loss point (Ψ_tlp_). After rehydration overnight, discs were sampled using a 6-mm-diameter punch from mature, healthy leaves collected on each branch, avoiding major and minor veins. Leaf discs wrapped in foil were frozen in liquid nitrogen for at least 2 minutes and then punctured 20 times quickly with sharp-tipped tweezers. Five repeat experiments using leaves from multiple individuals were carried out for every species at each site. The osmotic potential (Ψ_osm_) was measured with a VAPRO 5600 vapor pressure osmometer (Wescor, Logan, UT, USA) and Ψ_tlp_ (in MPa) was calculated as:

4 $${\Psi }_{tlp}=0.83{2\Psi }_{osm}-0.631$$

#### Morphometric traits

The morphometric trait data (Supplementary Table 2) were measured systematically by the same people (SPH and ICP) at all the sites. A standardized template for the field measurement of morphometric traits was used (Supplementary Table 5). This template provides a checklist of the traits and the categories used to describe them. The leaf traits assessed were texture, colour, size, thickness, orientation, display, shape, margin form, the presence of hairs, pubescence, pruinosity or rugosity, the presence of surface wax, hypostomatism, marginal curling (involute, revolute), smell (aromatic or fetid), the presence of a terminal notch or drip-tip, surface patterning, succulence, the presence and positioning of spines or thorns on the leaves. Illustrations of the various categories used in the classification of leaf margin and leaf shape are provided in supplementary materials, together with the template for leaf size categories (Supplementary Figs. 1–3). Although the distinction between spines and thorns is sometimes based on the source material (where thorns are derived from shoots and buds, and spines from any part of the leaf containing vascular material), here the differentiation is based on the shape of the protrusion (where thorns are triangular in shape and can be branched, and spines are unbranched and linear features). The checklist template also includes a limited amount of information on stem traits, such as form, colour, whether the stem is photosynthetic, the presence of stem hairs, pubescence, or pruinosity, and the presence of spines or thorns. For woody plants (trees, shrubs, climbers), the checklist also includes information on bark type (deciduous or not, with an indication of whether the bark is strip or chunk deciduous), the presence of furrowing, and also the presence of spines or thorns.

#### Plant Functional Types

The database includes information on life form, plant phenology, leaf form and leaf phenology (Table 8). Although these four pieces of information are used by many modellers in the definition of plant functional types (PFTs)^69,70^, they are not strictly species-specific traits. Thus, some species can occur as a tree, a small tree or a shrub (e.g. *Cyclobalanopsis obovatifolia*), or as a shrub or liana (e.g. *Smilax discotis*), depending on environmental conditions. Similarly, some species can behave as an evergreen or deciduous plant, depending on moisture availability (e.g. *Ulmus parvifolia*). Thus, this information is recorded for individual species at each site and no attempt was made to ensure that a given species was classified identically at all sites. In total 20 distinct life forms were recognized, including tree, small tree, low to high shrub, erect dwarf shrub, prostrate dwarf shrub, trailing shrub, liana, climber, forb, cushion forb, rosette forb, graminoid, bamboo, cycad, geophyte, stem succulent, succulent, pteridophyte, epiphyte, parasite. Plant phenology is recorded as perennial, biennial or annual. The primary distinction in leaf phenology is between deciduous and evergreen, but the classification used in the database also recognizes facultative deciduousness (semi-deciduous) and leaf-exchangers (i.e. plants that retain their leaves for nearly the whole year but drop and replace all of the leaves in a single short period, rather than replacing some leaves continuously through the year as evergreens do). The concept of leaf phenology is only relevant for woody plants (trees, shrubs, lianas) and so is not recorded for e.g. forbs or climbers.

#### Vegetation

The local vegetation was not recorded in the field at each site, and in any case such descriptions are hard to standardize. The CPTDv2 database contains information on vegetation type extracted from the digital vegetation map of China at the scale of 1:1 million^71^, which uses 55 plant communities (48 natural plant communities and seven cropping systems). CPTDv2 further provides information on vegetation clusters aggregated from those fundamental plant communities from the Vegetation Atlas of China based on their bioclimatic context^72^. CPTDv2 also contains information on potential natural vegetation (PNV), derived from an updated version of the^73^ global mapping of PNV. This PNV map was produced using pollen-based vegetation reconstructions as a target, a set of 160 spatially explicit co-variate data sets representing the climatic, topographic, geologic, and hydrological controls on plant growth and survival, and an ensemble machine-learning approach to account for the relationships between vegetation types and these covariates (Table 9). The original version of the map had a spatial resolution of 1 km; the updated version used here (https://github.com/Envirometrix/PNVmaps) has a resolution of 250 m.

#### Climate

Climatological estimates of monthly temperature, precipitation and fraction of sunshine hours were derived from records from 1814 meteorological stations (740 stations have observations from 1971 to 2000, the rest from 1981 to 1990: China Meteorological Administration, unpublished data), interpolated to a 0.01 grid using a three-dimensional thin-plate spline (ANUSPLIN version 4.36;^74^. These monthly climatological data were used directly to calculate the mean temperature of the coldest month (MTCO), mean annual temperature (MAT), mean monthly precipitation (MMP) and mean annual precipitation (MAP). Bioclimatic variables at each site were calculated from the interpolated monthly temperature, precipitation and fraction of sunshine hours using the Simple Process-Led Algorithms for Simulating Habitats (SPLASH) model^75^. The bioclimatic variables include total annual photosynthetically active radiation during the growing season when mean daily temperatures are >0 °C (PAR0), the daily mean photosynthetically active radiation during the growing season (mPAR0), growing degree days above a baseline of 0 °C (GDD0), the daily mean temperature during the growing season (mGDD0), the ratio of actual to equilibrium evapotranspiration (α), and a moisture index (MI) defined as the ratio of mean annual precipitation to potential evapotranspiration. We also calculated the timing of peak rainfall and rainfall seasonality, using metrics described in Kelley, *et al*.^76^ (Supplementary Table 3).

The topography in the Gongga region is complex, and the standard climate data set is inadequate to capture the elevation impacts of local climate at the sites there^13^. We therefore also provide alternative estimates of climatic variables for the Gongga elevation transects using 17 weather stations from the region with records from January 2017 to December 2019 (Supplementary Table 4). These 17 stations range in elevation from 422 m to 3951 m, in latitude from 28° to 31° N, and in longitude from 99.1° to 103.8° E. The climatological records for each station were downloaded from China Meteorological Data Service Centre, National Meteorological Information Centre (http://data.cma.cn/data/detail/dataCode/A.0012.0001.html). The monthly maximum and minimum temperature, precipitation, percentage of possible sunshine hours were extracted. The monthly mean temperature was calculated as the average of maximum and minimum temperature. The elevationally-sensitive ANUSPLIN interpolation scheme^74^ was used to provide estimates of meteorological variables at each site as described above. The bioclimatic variables were calculated following the same methodology as the 0.01 grid data described above.

#### Soil

Soil was not sampled in the field, but to facilitate analyses we provide soil information extracted from the Harmonized World Soil Database (HWSD) v1.2^77^ (Table 10). The HWSD v1.2 is a high-resolution (0.05°) soil database with soil characteristics determined from real soil profiles. The soil properties were estimated in a harmonized way, where the actual soil profile data and the development of pedotransfer rules were undertaken in cooperation with ISRIC and ESBN drawing on the WISE soil profile database and some earlier works^78,79^. The HWSD v1.2 provides information for the uppermost soil layer (0–30 cm) and the deeper soil layer (30–100 cm). Although HWSD v1.2 contains information on a large number of soil properties, we only extracted information on soil texture (sand fraction, silt fraction and clay fraction), the content of organic carbon, soil pH in water, and cation exchange capacity.

## Data Records

The database is available from figshare^80^. The database link is: https://figshare.com/articles/dataset/The_China_Plant_Trait_Database_Version_2_0/19448219. An overview of the data files, definitions, formats and a summary of the variations for each variable (when applicable) are given in the series of tables below (Tables 1–10, Supplementary Tables 1–4).

## Technical Validation

### Trait data validation

Most of the data in the China Plant Trait Database were provided by the authors. 26 out of 140 total sites were sampled by the same team and following standardized measurement protocols. Although the morphological trait measurements are subjective, these assessments were made in the field by the same two people (ICP, SPH) using a standardized reporting sheet (Supplementary Table 5) and thus is consistent between sites. 18 sites were extracted from the literature, but only in cases where the publication provided both an adequate description of the sampling protocol and methods, the individual sites could be accurately located, and where the primary data were provided.

Quality control procedures were applied to ensure that units were reported correctly. We checked for inconsistencies between different measurements, including e.g. comparing scanned measurements of leaf area and field-based CLAMP classifications of leaf area (Supplementary Fig. 3). The data for each trait was examined for abnormal values or outliers. In most cases, these issues could be resolved by checking field records or original data sheets. In a few cases, these inconsistencies and/or errors were present in the field or laboratory records – these doubtful measurements have been moved in the database. Some of the measurements of chemical and photosynthetic traits are far outside the typical observed range according to the China Plant Trait Database, or other global datasets^1,81,82^ but are not due to recording errors; these measurements have been flagged in the database as potentially unreliable. The criteria for outlier flags are summarized in Table 11 with visualized plots shown in Supplementary Fig. 4.

**Table 11:** Summary on traits flagged as outliers.

Traits	Number of outliers	Plot-based threshold	Dataset-based threshold
LA	7		<0.79 mm^2^ (broadleaved)
SLA	144		>204 m^2^/kg
LMA	144		<0.0049 kg/m^2^
LDMC	1	>1000 mg/g	
C_mass_	33		<250, >700 g/kg
N_mass_	13		<2.5, >69 g/kg
P_mass_	7		<0.1 g/kg
K_mass_	1	>90 g/kg	
N_area_	124	<0.11, >8.8 g/m^2^	
P_area_	113	<0.00024, >0.96 g/m^2^	
K_area_	51	<0.003, >7.3 g/m^2^	
Amax_Photo	6	<0, >60 umol/m^2^/s	
Amax_Gs	12	<0, >3 mol/m^2^/s	
Amax_Ci:Ca	5	<0, >1	
Amax_E	6	<0 mmol/m^2^/s	
Asat_Photo	2	<0, >50 umol/m^2^/s	
Asat_Gs	15	<0, >3 mol/m^2^/s	
Asat_Ci:Ca	8	<0, >1	
Asat_E	5	<0 mmol/m^2^/s	
*V*_cmax_	5		<0, >180 umol/m^2^/s
*Jmax*	1	>500 umol/m^2^/s	
Fv:Fm	5	<0.5, >0.9	

## Usage Notes

When using the data set, we kindly request that you cite this article, recognizing the hard work that went into collecting the data and the authors’ willingness to make it publicly available.

## Supplementary information

Supplementary information

## Data Availability

The R code for estimating photosynthetic capacities, calculating the timing and seasonality of precipitation, and extracting soil and vegetation information are available in the open GitHuB repository (https://github.com/lpice/code-CPTDv2-.git) The SPLASH code, in four programming languages (C++, FOR- TRAN, Python, and R), is available on an online repository under the GNU Lesser General Public License (https://bitbucket.org/labprentice/splash)
