# Supplementary material for: The China plant trait database version 2
Source: Sci Data. 2022 Dec 15;9:769. doi: 10.1038/s41597-022-01884-4 (PMC9755148; doi:10.1038/s41597-022-01884-4)

**Supplementary information of The China plant trait database version 2**

**Supplementary Tables 1-5**

**Supplementary Figures 1-4**

**Supplementary Table 1: Photosynthetic traits**

| **Field name** | **Definition** | **Units** | **Number of observations** | **Min** | **Median** | **Max** |
| --- | --- | --- | --- | --- | --- | --- |
| Sample id | unique identifier for each sample | NA | 1505 |  |  |  |
| Amax_Photo | rate of photosynthesis under light and CO2 saturation | umol/m2/s | 826 | 0.98 | 14.73 | 58.55 |
| Amax_Gs | stomatal conductance to water at which Amax was measured | mol/m2/s | 826 | 0.0043 | 0.10 | 2.44 |
| Amax_Ci:Ca | ratio of internal to external CO2 when Amax was measured | unitless | 826 | 0.14 | 0.84 | 0.99 |
| Amax_E | transpiration rate when Amax was measured | mmol/m2/s | 826 | 0.00057 | 1.57 | 12.66 |
| Amax_VPD | the vapour pressure deficit at which Amax was measured | kpa | 826 | 0.12 | 1.23 | 3.01 |
| Amax_Tleaf | the temperature at which Amax was measured | ˚C | 826 | 11.88 | 24.92 | 36.00 |
| Amax_CO2 | the CO2 level at which Amax was measured | ppm | 826 | 1454.80 | 1957.73 | 2002.79 |
| Asat_Photo | rate of photosynthesis under light saturation | umol/m2/s | 1020 | 0.20 | 7.19 | 47.13 |
| Asat_Gs | stomatal conductance to water at which Asat was measured | mol/m2/s | 801 | 0.0049 | 0.14 | 2.46 |
| Asat_Ci:Ca | ratio of internal to external CO2 when Asat was measured | unitless | 801 | 0.24 | 0.78 | 0.99 |
| Asat_E | transpiration rate when Asat was measured | mmol/m2/s | 801 | 0.28 | 1.97 | 13.33 |
| Asat_VPD | the vapour pressure deficit at which Amax was measured | kpa | 801 | 0.14 | 1.20 | 3.02 |
| Asat_Tleaf | the temperature at which Asat was measured | ˚C | 977 | 11.17 | 25.00 | 33.27 |
| Asat_CO2 | the CO2 level at which Asat was measured | ppm | 976 | 371.70 | 393.96 | 404.69 |
| Vcmax | the maximum capacity of carboxylation | umol/m2/s | 981 | 0.43 | 31.21 | 174.63 |
| Jmax | the maximum capacity of electron-transport | umol/m2/s | 826 | 5.11 | 69.62 | 462.46 |
| Fv:Fm | potential rate of photosynthetic electron transport as measured by chlorophyll fluorescence | unitless | 475 | 0.50 | 0.79 | 0.87 |
| QY | actual rate of photosynthetic electron transport as measured by chlorophyll fluorescence | umol/m2/s | 477 | 0.14 | 0.73 | 0.82 |
| flagged | traits with some potential problems or unrealistic values for each sample |  |  |  |  |  |

**Supplementary Table 2: Morphometric traits**

| **Field name** | **Definition** | **Category** | **Number of observations** |
| --- | --- | --- | --- |
| Sample ID | unique identifier for each sample | NA | 2415 |
| Leaf texture | description of the texture of a leaf, particularly as related to flexibility and/or toughness as distinct from surface characteristics | fleshy | 2336 |
|  |  | papery |  |
|  |  | malacophyll |  |
|  |  | leathery |  |
|  |  | coriaceous |  |
|  |  | rigidly coriaceous |  |
| Leaf colour -adaxial | the colour of the upper surface of the leaf, i.e. the surface facing the stem | bright green | 2356 |
|  |  | green |  |
|  |  | dark green |  |
|  |  | mottled green |  |
|  |  | pale green |  |
|  |  | glaucous |  |
|  |  | yellow-green |  |
|  |  | grey-green |  |
|  |  | yellow |  |
|  |  | silvery-grey |  |
| Leaf colour - abaxial | the colour of the lower surface of the leaf, i.e. the surface not facing the stem | bright green | 2357 |
|  |  | green |  |
|  |  | dark green |  |
|  |  | mottled green |  |
|  |  | pale green |  |
|  |  | brown-green |  |
|  |  | glaucous |  |
|  |  | yellow-green |  |
|  |  | grey-green |  |
|  |  | brown |  |
|  |  | yellow-brown |  |
|  |  | pale brown |  |
|  |  | purple |  |
|  |  | reddish-green |  |
|  |  | white |  |
|  |  | yellow |  |
|  |  | silvery-grey |  |
| Leaf size | categorical classification of typical leaf size as estimated using the modified CLAMP scheme | pico | 2330 |
|  |  | lepto |  |
|  |  | nano |  |
|  |  | micro |  |
|  |  | noto |  |
|  |  | meso |  |
|  |  | macro |  |
| Leaf thickness | categorical classification of typical leaf thickness, approximately measured | thick (>2mm) | 2274 |
|  |  | medium (0.5-2mm) |  |
|  |  | thin (<0.5mm) |  |
| Leaf orientation | categorical description of the angle of the individual leaf with respect to the stem | erect | 2316 |
|  |  | semi-erect |  |
|  |  | patent |  |
|  |  | pendulous |  |
|  |  | reclinate |  |
| Leaf display | organisation of leaves within the individual plant canopy | 2D | 2303 |
|  |  | 3D |  |
| Leaf shape | description of the shape of the leaf blade (or leaflet in the case of compound leaves) shape | acicular | 2204 |
|  |  | cordate |  |
|  |  | deltoid |  |
|  |  | elliptic |  |
|  |  | falcate |  |
|  |  | hastate |  |
|  |  | lanceolate |  |
|  |  | linear |  |
|  |  | lyrate |  |
|  |  | obcordate |  |
|  |  | oblanceolate |  |
|  |  | oblong |  |
|  |  | obovate |  |
|  |  | orbicular |  |
|  |  | oval |  |
|  |  | ovate |  |
|  |  | reniform |  |
|  |  | runcinate |  |
|  |  | sagittate |  |
|  |  | spatulate |  |
|  |  | cordate-lanceolate |  |
|  |  | triangular |  |
|  |  | elliptic-lanceolate |  |
|  |  | linear-lanceolate |  |
|  |  | ovate-lanceolate |  |
|  |  | obovate-lanceolate |  |
|  |  | ovate-bipinnate |  |
|  |  | fishtail |  |
|  |  | palmate |  |
|  |  | palmatifid |  |
|  |  | pinnatifid |  |
|  |  | rhomboid |  |
|  |  | tulip-shaped |  |
|  |  | trilobite |  |
|  |  | quinquelobate |  |
|  |  | septemlobate |  |
| Leaf margin | description of the nature of the margin of the leaf of leaflet | entire | 2277 |
|  |  | finely toothed |  |
|  |  | toothed |  |
|  |  | crenate |  |
|  |  | crenulate |  |
|  |  | lobed |  |
|  |  | dissected |  |
|  |  | highly dissected |  |
|  |  | incised |  |
| Leaf hairs | indication of presence or absence of hairs on the leaf; if hairs are present, the location is also recorded | adaxial | 2260 |
|  |  | abaxial |  |
|  |  | on midrib or veins |  |
|  |  | marginal |  |
|  |  | basal |  |
|  |  | no |  |
| Leaf pubescence | indication of presence or absence of very fine hairs or pubescence on the leaf; if present, the location is also recorded | adaxial | 2260 |
|  |  | abaxial |  |
|  |  | on midrib or veins |  |
|  |  | marginal |  |
|  |  | no |  |
| Leaf pruinosity | indication of presence or absence of a bloom or powdery secretion that can be removed mechanically on the leaf; if present, the location is also recorded | adaxial | 2260 |
|  |  | abaxial |  |
|  |  | on midrib or veins |  |
|  |  | no |  |
| Leaf rugose | indication of presence or absence of surface roughness caused by surface protrusions on a leaf; if present, the location is also recorded | adaxial | 2282 |
|  |  | abaxial |  |
|  |  | on midrib or veins |  |
|  |  | no |  |
| Leaf waxy | indication of whether there is a continuous waxy deposit on the surface of the leaf. A distinction was made in the field between waxy and glossy surfaces, presumed to reflect differences in the structure of this epicuticular layer | yes | 2282 |
|  |  | glossy |  |
|  |  | no |  |
| Leaf hypostomatic | indication of whether stomata were present only on the abaxial side of the leaf | yes | 2282 |
|  |  | no |  |
| Leaf revolute | indication of whether the leaf margin was curled downwards toward the underside of the leaf. A distinction was made between leaves that showed slight or pronounced curling | yes | 2282 |
|  |  | slightly |  |
|  |  | no |  |
| Leaf involute | indication of whether the leaf margin was curled toward the upperside of the leaf. A distinction was made between leaves that showed slight or pronounced curling | yes | 2282 |
|  |  | slightly |  |
|  |  | no |  |
| Leaf aromatic | indication of whether the leaves contain aromatic compounds, assessed from the smell of the leaves when broken in the field | yes | 2282 |
|  |  | no |  |
| Leaf fetid | indication of whether the leaves have a rank or unpleasant smell when broken in the field | yes | 2282 |
|  |  | no |  |
| Leaf driptip | presence of an elongated, downward oriented extension at the tip of the leaf or leaflet blade, assumed to relate to removal of excess water | yes | 2282 |
|  |  | no |  |
| Leaf terminal notch | presence of a notch or narrow cleft at the tip of the leaf or leaflet blade | Yes | 2282 |
|  |  | no |  |
| Leaf surface patterning | leaves with marked surface structures | yes | 2282 |
|  |  | no |  |
|  |  | slight |  |
| Leaf succulence | indication of whether the leaf stores water, assessed from whether the leaves are thick and fleshy and whether water is released when the leaf is broken. A distinction is made in the field between slightly succulent (swollen) leaves and truly succulent leaves | yes | 2282 |
|  |  | no |  |
| Leaf spines | presence of leaf spines; if spines are present, the location is also recorded | no | 2284 |
|  |  | adaxial |  |
|  |  | abaxial |  |
|  |  | on midrib or veins |  |
|  |  | marginal |  |
|  |  | terminal |  |
| Leaf thorns | presence of leaf thorns | no | 2284 |
|  |  | yes |  |
| Stem form | description of the appearance of the stem, in terms of shape, and/or the presence of protuberances, attachments, or markings | non-distinctive | 2153 |
|  |  | triangular |  |
|  |  | quadrangular |  |
|  |  | hectangular |  |
|  |  | winged |  |
|  |  | ridged |  |
|  |  | corky |  |
|  |  | leaves attached directly |  |
|  |  | deciduous sheath |  |
|  |  | white lines |  |
|  |  | white spots |  |
| Stem colour | description of the base colour of the stem | black | 2152 |
|  |  | dark brown |  |
|  |  | brown |  |
|  |  | pale brown |  |
|  |  | green-brown |  |
|  |  | grey-brown |  |
|  |  | red-brown |  |
|  |  | purple-brown |  |
|  |  | dark green |  |
|  |  | green |  |
|  |  | pale green |  |
|  |  | green-purple |  |
|  |  | yellow-green |  |
|  |  | red-green |  |
|  |  | glaucous |  |
|  |  | silver-grey |  |
|  |  | grey |  |
|  |  | yellow |  |
|  |  | purple |  |
|  |  | red |  |
| Stem photo | indication of whether the stem is photosynthetic or not | yes | 2153 |
|  |  | no |  |
| Stem hairy | indication of presence of hairs on the stem; if present, the density is also recorded | yes | 2152 |
|  |  | no |  |
|  |  | finely |  |
| Stem pubescent | indication of presence of very fine hairs or pubescence on the stem; if present, the density is also records | yes | 2151 |
|  |  | no |  |
|  |  | finely |  |
| Stem pruinose | indication of presence of a bloom or powdery secretion that can be removed mechanically on the leaf | yes | 2152 |
|  |  | no |  |
| Stem rugose | indication of presence of a rough surface caused by protuberances | yes | 1860 |
|  |  | no |  |
| Stem succulent | indication of presence of water-retention in the stem | yes | 2152 |
|  |  | no |  |
| Stem spines | presence of spines on the stem | yes | 2152 |
|  |  | no |  |
| Stem thorns | presence of thorns on the stem | yes | 2152 |
|  |  | no |  |
| Bark deciduous | indication of whether the bark is shed on a regular basis; bark shedding as a result of specific damage (e.g. insect attack, fire damage) is not taken into consideration. If the bark is deciduous, the way in which bark is detached is recorded | non-deciduous | 984 |
|  |  | chunk |  |
|  |  | strip |  |
|  |  | fissured |  |
| Spines elsewhere | presence of spines on the trunk or major branches | yes | 2142 |
|  |  | no |  |

**Supplementary Table 3: High-resolution climate**

| **Field name** | **Definition** | **Units** |
| --- | --- | --- |
| Site ID | unique identifier for each site | NA |
| Lat_grid | latitude of the extracted 1 km gridded cell | decimal degrees |
| Lon_grid | longitude of the extracted 1 km gridded cell | decimal degrees |
| Temp Jan | mean January temperature as obtained from the 1 km gridded climatology | ˚C |
| Temp Feb | mean February temperature as obtained from the 1 km gridded climatology | ˚C |
| Temp Mar | mean March temperature as obtained from the 1 km gridded climatology | ˚C |
| Temp April | mean April temperature as obtained from the 1 km gridded climatology | ˚C |
| Temp May | mean May temperature as obtained from the 1 km gridded climatology | ˚C |
| Temp June | mean June temperature as obtained from the 1 km gridded climatology | ˚C |
| Temp July | mean July temperature as obtained from the 1 km gridded climatology | ˚C |
| Temp Aug | mean August temperature as obtained from the 1 km gridded climatology | ˚C |
| Temp Sep | mean September temperature as obtained from the 1 km gridded climatology | ˚C |
| Temp Oct | mean October temperature as obtained from the 1 km gridded climatology | ˚C |
| Temp Nov | mean November temperature as obtained from the 1 km gridded climatology | ˚C |
| Temp Dec | mean December temperature as obtained from the 1 km gridded climatology | ˚C |
| Prec Jan | mean January precipitation as obtained from the 1 km gridded climatology | mm |
| Prec Feb | mean February precipitation as obtained from the 1 km gridded climatology | mm |
| Prec Mar | mean March precipitation as obtained from the 1 km gridded climatology | mm |
| Prec April | mean April precipitation as obtained from the 1 km gridded climatology | mm |
| Prec May | mean May precipitation as obtained from the 1 km gridded climatology | mm |
| Prec June | mean June precipitation as obtained from the 1 km gridded climatology | mm |
| Prec July | mean July precipitation as obtained from the 1 km gridded climatology | mm |
| Prec Aug | mean August precipitation as obtained from the 1 km gridded climatology | mm |
| Prec Sep | mean September precipitation as obtained from the 1 km gridded climatology | mm |
| Prec Oct | mean October precipitation as obtained from the 1 km gridded climatology | mm |
| Prec Nov | mean November precipitation as obtained from the 1 km gridded climatology | mm |
| Prec Dec | mean December precipitation as obtained from the 1 km gridded climatology | mm |
| Sunh Jan | mean January sunshine hours as obtained from the 1 km gridded climatology and expressed as a percentage of total possible sunshine hours | % |
| Sunh Feb | mean February sunshine hours as obtained from the 1 km gridded climatology and expressed as a percentage of total possible sunshine hours | % |
| Sunh Mar | mean March sunshine hours as obtained from the 1 km gridded climatology and expressed as a percentage of total possible sunshine hours | % |
| Sunh April | mean April sunshine hours as obtained from the 1 km gridded climatology and expressed as a percentage of total possible sunshine hours | % |
| Sunh May | mean May sunshine hours as obtained from the 1 km gridded climatology and expressed as a percentage of total possible sunshine hours | % |
| Sunh June | mean June sunshine hours as obtained from the 1 km gridded climatology and expressed as a percentage of total possible sunshine hours | % |
| Sunh July | mean July sunshine hours as obtained from the 1 km gridded climatology and expressed as a percentage of total possible sunshine hours | % |
| Sunh Aug | mean August sunshine hours as obtained from the 1 km gridded climatology and expressed as a percentage of total possible sunshine hours | % |
| Sunh Sep | mean September sunshine hours as obtained from the 1 km gridded climatology and expressed as a percentage of total possible sunshine hours | % |
| Sunh Oct | mean October sunshine hours as obtained from the 1 km gridded climatology and expressed as a percentage of total possible sunshine hours | % |
| Sunh Nov | mean November sunshine hours as obtained from the 1 km gridded climatology and expressed as a percentage of total possible sunshine hours | % |
| Sunh Dec | mean December sunshine hours as obtained from the 1 km gridded climatology and expressed as a percentage of total possible sunshine hours | % |
| MTCO | mean temperature of the coldest month | ˚C |
| MAT | mean annual temperature | ˚C |
| MI | ratio of annual actual evapotranspiration to annual precipitation | unitless |
| alpha | ratio of actual to equilibrium evapotranspiration | unitless |
| GDD0 | growing degree days above a baseline of 0°C | ˚C days |
| mGDD0 | daily mean temperature during the growing season when temperatures are >0°C | ˚C |
| PAR0 | total annual photosynthetically active radiation | mol photon m^–2^ |
| mPAR0 | daily mean photosynthetically active radiation during the growing season when temperatures are >0°C | mol photon m^–2^ |
| Prec timing | the timing of peak precipitation, expressed as a vector where January 1^st^ is arbitrarily set to an angle of 0° | unitless |
| Prec season | the seasonality of precipitation, where 0 means that precipitation is equally distributed in every month of the year and 1 means that precipitation is concentrated in one month of the year | unitless |
| MMP | mean monthly precipitation | mm |
| MAP | mean annual precipitation | mm |

**Supplementary Table 4: Gongga local climate**

| **Field name** | **Definition** | **Units** |
| --- | --- | --- |
| Site ID | unique identifier for each site | NA |
| Temp Jan | mean January temperature obtained by extrapolating from local meteorological stations and flux sites | ˚C |
| Temp Feb | mean February temperature as obtained from the 1 km gridded climatology | ˚C |
| Temp Mar | mean March temperature obtained by extrapolating from local meteorological stations and flux sites | ˚C |
| Temp April | mean April temperature obtained by extrapolating from local meteorological stations and flux sites | ˚C |
| Temp May | mean May temperature obtained by extrapolating from local meteorological stations and flux sites | ˚C |
| Temp June | mean June temperature obtained by extrapolating from local meteorological stations and flux sites | ˚C |
| Temp July | mean July temperature obtained by extrapolating from local meteorological stations and flux sites | ˚C |
| Temp Aug | mean August temperature obtained by extrapolating from local meteorological stations and flux sites | ˚C |
| Temp Sep | mean September temperature obtained by extrapolating from local meteorological stations and flux sites | ˚C |
| Temp Oct | mean October temperature obtained by extrapolating from local meteorological stations and flux sites | ˚C |
| Temp Nov | mean November temperature obtained by extrapolating from local meteorological stations and flux sites | ˚C |
| Temp Dec | mean December temperature obtained by extrapolating from local meteorological stations and flux sites | ˚C |
| Prec Jan | mean January precipitation obtained by extrapolating from local meteorological stations and flux sites | mm |
| Prec Feb | mean February precipitation obtained by extrapolating from local meteorological stations and flux sites | mm |
| Prec Mar | mean March precipitation obtained by extrapolating from local meteorological stations and flux sites | mm |
| Prec April | Mean April precipitation obtained by extrapolating from local meteorological stations and flux sites | mm |
| Prec May | mean May precipitation obtained by extrapolating from local meteorological stations and flux sites | mm |
| Prec June | mean June precipitation obtained by extrapolating from local meteorological stations and flux sites | mm |
| Prec July | mean July precipitation obtained by extrapolating from local meteorological stations and flux sites | mm |
| Prec Aug | Mean August precipitation obtained by extrapolating from local meteorological stations and flux sites | mm |
| Prec Sep | mean September precipitation obtained by extrapolating from local meteorological stations and flux sites | mm |
| Prec Oct | mean October precipitation obtained by extrapolating from local meteorological stations and flux sites | mm |
| Prec Nov | mean November precipitation obtained by extrapolating from local meteorological stations and flux sites | mm |
| Prec Dec | mean December precipitation obtained by extrapolating from local meteorological stations and flux sites | mm |
| Sunh Jan | mean January sunshine hours obtained by extrapolating from local meteorological stations and flux sites and expressed as a percentage of total possible sunshine hours | % |
| Sunh Feb | mean February sunshine hours obtained by extrapolating from local meteorological stations and flux sites and expressed as a percentage of total possible sunshine hours | % |
| Sunh Mar | mean March sunshine hours obtained by extrapolating from local meteorological stations and flux sites and expressed as a percentage of total possible sunshine hours | % |
| Sunh April | mean April sunshine hours obtained by extrapolating from local meteorological stations and flux sites and expressed as a percentage of total possible sunshine hours | % |
| Sunh May | mean May sunshine hours obtained by extrapolating from local meteorological stations and flux sites and expressed as a percentage of total possible sunshine hours | % |
| Sunh June | mean June sunshine hours obtained by extrapolating from local meteorological stations and flux sites and expressed as a percentage of total possible sunshine hours | % |
| Sunh July | mean July sunshine hours obtained by extrapolating from local meteorological stations and flux sites and expressed as a percentage of total possible sunshine hours | % |
| Sunh Aug | mean August sunshine hours obtained by extrapolating from local meteorological stations and flux sites and expressed as a percentage of total possible sunshine hours | % |
| Sunh Sep | mean September sunshine hours obtained by extrapolating from local meteorological stations and flux sites and expressed as a percentage of total possible sunshine hours | % |
| Sunh Oct | mean October sunshine hours obtained by extrapolating from local meteorological stations and flux sites and expressed as a percentage of total possible sunshine hours | % |
| Sunh Nov | mean November sunshine hours obtained by extrapolating from local meteorological stations and flux sites and expressed as a percentage of total possible sunshine hours | % |
| Sunh Dec | mean December sunshine hours obtained by extrapolating from local meteorological stations and flux sites and expressed as a percentage of total possible sunshine hours | % |
| MTCO | mean temperature of the coldest month | ˚C |
| MAT | mean annual temperature | ˚C |
| MI | ratio of annual actual evapotranspiration to annual precipitation | unitless |
| alpha | ratio of actual to equilibrium evapotranspiration | unitless |
| GDD0 | growing degree days above a baseline of 0°C | ˚C days |
| mGDD0 | daily mean temperature during the growing season when temperatures are >0°C | ˚C |
| PAR0 | total annual photosynthetically active radiation | mol photon m^–2^ |
| mPAR0 | daily mean photosynthetically active radiation during the growing season when temperatures are >0°C | mol photon m^–2^ |
| Prec timing | the timing of peak precipitation, expressed as a vector where January 1^st^ is arbitrarily set to an angle of 0° | unitless |
| Prec season | the seasonality of precipitation, where 0 means that precipitation is equally distributed in every month of the year and 1 means that precipitation is concentrated in one month of the year | unitless |
| MMP | mean monthly precipitation | mm |
| MAP | mean annual precipitation | mm |

**Supplementary Table 5** The crib sheet used for recording leaf morphological traits in the field

| Site No: | Site name: | | | | |
| --- | --- | --- | --- | --- | --- |
| Elev: | **Lat:** | | | **Long:** | |
| Species: | | | | | |
| Gymnosperm yes/no | | **Monocot** yes/no | | | |
| Life form | *tree, small tree,*  *low to high shrub, erect dwarf shrub, prostrate dwarf shrub, trailing shrub, liana, climber,*  *forb, cushion forb, rosette forb, graminoid, bamboo,*  *cycad, geophyte, stem succulent, succulent,*  *pteridophyte, epiphyte, parasite* | | | | |
| Plant phenology | *annual, biennial, perennial* | | | | |
| Leaf type | *aphyllous, broad, needle, scale* | | | | |
| Leaf phenology  (only woody plants) | *evergreen, deciduous, semi-deciduous, leaf-exchanger* | | | | |
| Leaf texture | *fleshy, papery, malacophyll, leathery, coriaceous, rigidly coriaceous* | | | | |
| Leaf colour- adaxial  (upper surface) | *bright green, green, dark green, mottled green, pale green,*  *glaucous, yellow-green, yellow, silvery-grey, grey-green* | | | | |
| Leaf colour- abaxial  (lower surface) | *bright green, green, dark green, mottled green, pale green, brown-green, reddish-green, glaucous, yellow-green, yellow-brown, pale brown, brown, purple, white, yellow, silvery-grey, grey-green* | | | | |
| Leaf size | pico (<5mm^2^), lepto (5-25mm^2^), nano (25-250mm^2^), micro (250-2000mm^2^), noto (2000-4500mm^2^), meso (4500-20000mm^2^), macro (20000-150000mm^2^) | | | | |
| Leaf thickness | *Thick, medium, thin* | | | | |
| Leaf orientation | *erect, semi-erect, patent, pendulous, reclinate* | | | | |
| Leaf display | 2D, 3D | | | | |
| Leaf shape  (nb describes overall outline of leaf) | *Acicular, cordate, deltoid, elliptic, falcate, hastate, lanceolate, linear, lyrate, obcordate, oblanceolate, oblong, obovate, orbicular, oval, ovate, reniform, runcinate, sagittate, spatulate, triangular*  *cordate-lanceolate, elliptic-lanceolate, linear-lanceolate, ovate-lanceolate,* *ovate-bipinnate,*  *obovate-lanceolate, fishtail,*  *palmate, palmatifid, pinnatifid, rhomboid, tulip-shaped, trilobate, quinquelobate, septemlobate* | | | | |
| Leaf margin | *entire, finely toothed, toothed, crenate, crenulate, lobed, dissected,*  *highly dissected, incised* | | | | |
| Leaf hairiness | *adaxial, abaxial, midrib/veins, marginal, basal* NO | | | | |
| Leaf pubescent | *adaxial, abaxial, midrib/veins, marginal* NO | | | | |
| Leaf pruinose | *adaxial, abaxial, midrib/veins*  NO | | | | |
| Leaf rugose | *adaxial, abaxial, midrib/veins*  NO | | | | |
| Leaf waxy | Yes No Glossy | | | | |
| Leaf hypostomatic | Yes No | | | | |
| Leaf revolute  (turned. under, ∩) | Yes No Slightly | | | | |
| Leaf involute  (turned upwards, ∪) | Yes No Slightly | | | | |
| Leaf aromatic | Yes No | | | | |
| Leaf fetid | Yes No | | | | |
| Leaf drip-tip | Yes No | | | | |
| Leaf terminal notch | Yes No | | | | |
| Leaf surface patterning | Yes No Slight | | | | |
| Leaf succulence | Yes No | | | | |
| Leaf spines | *adaxial, abaxial, midrib/veins, marginal, terminal* NO | | | | |
| Leaf thorn | Yes No | | | | |
|  |  | | | | |
| Stem form | *non-distinctive*, *triangular, quadrangular, hectangular, winged*, *ridged,* *corky*, *leaves attached directly*, *deciduous sheath, white lines*, *white spots* | | | | |
| Stem colour | *black, dark brown, brown, pale brown, green-brown, grey-brown,*  *red-brown, dark green, green, pale green, green-purple, yellow-green, purple-brown,*  *red-green, glaucous, silver-grey, grey, yellow, purple, red* | | | | |
| Stem photo | Yes No | | | | |
| Stem hairy | Yes No finely | | | | |
| Stem pubescent | Yes No finely | | | | |
| Stem pruinose | Yes No | | | | |
| Stem succulence | Yes No | | | | |
| Stem spiny | Yes No | | Spines elsewhere | | Yes No |
| Stem thorns | Yes No | | Thorns elsewhere | | Yes No |
| Bark | non-deciduous, chunk, strip, fissured | | | | |

**Supplementary Figure 1: The illustrating examples of leaf marginal types.** Noticably, the following types are described in the crib sheet differently: combine serrate, doubly serrate, dentate (= toothed), denticulate and serrulate (= finely toothed), sinuate (= crenate), lacerate (= dissected), laciniate (= highly dissected). Cliniate, involute and involute are not recorded as leaf marginal trait in the crib sheet.


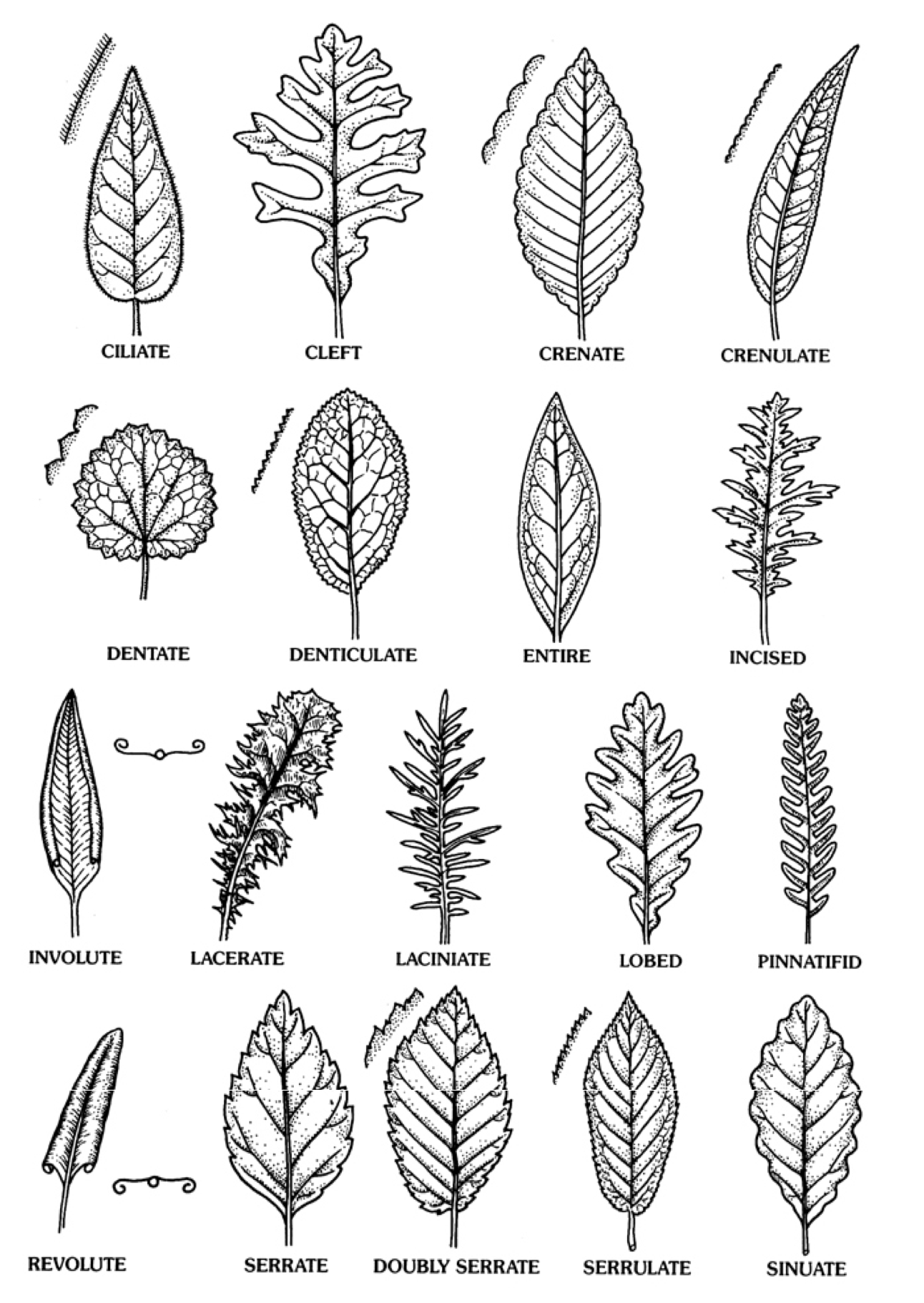


**Supplementary Figure 2: The illustrating examples of leaf shape types.**


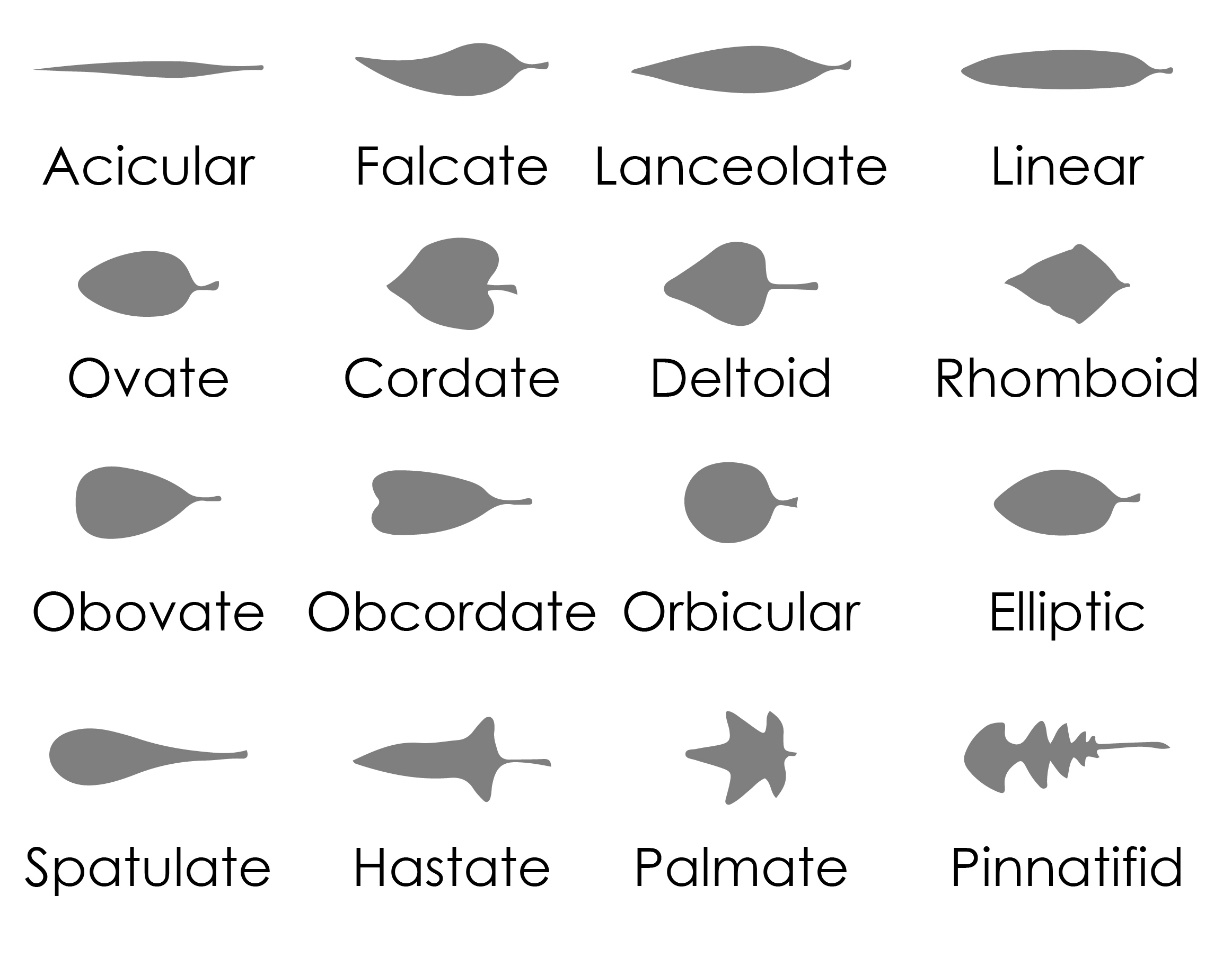


**Supplementary Figure 3: The CLAMP leaf size template (http://clamp.ibcas.ac.cn/CLAMP_Leaf_Size.html)**


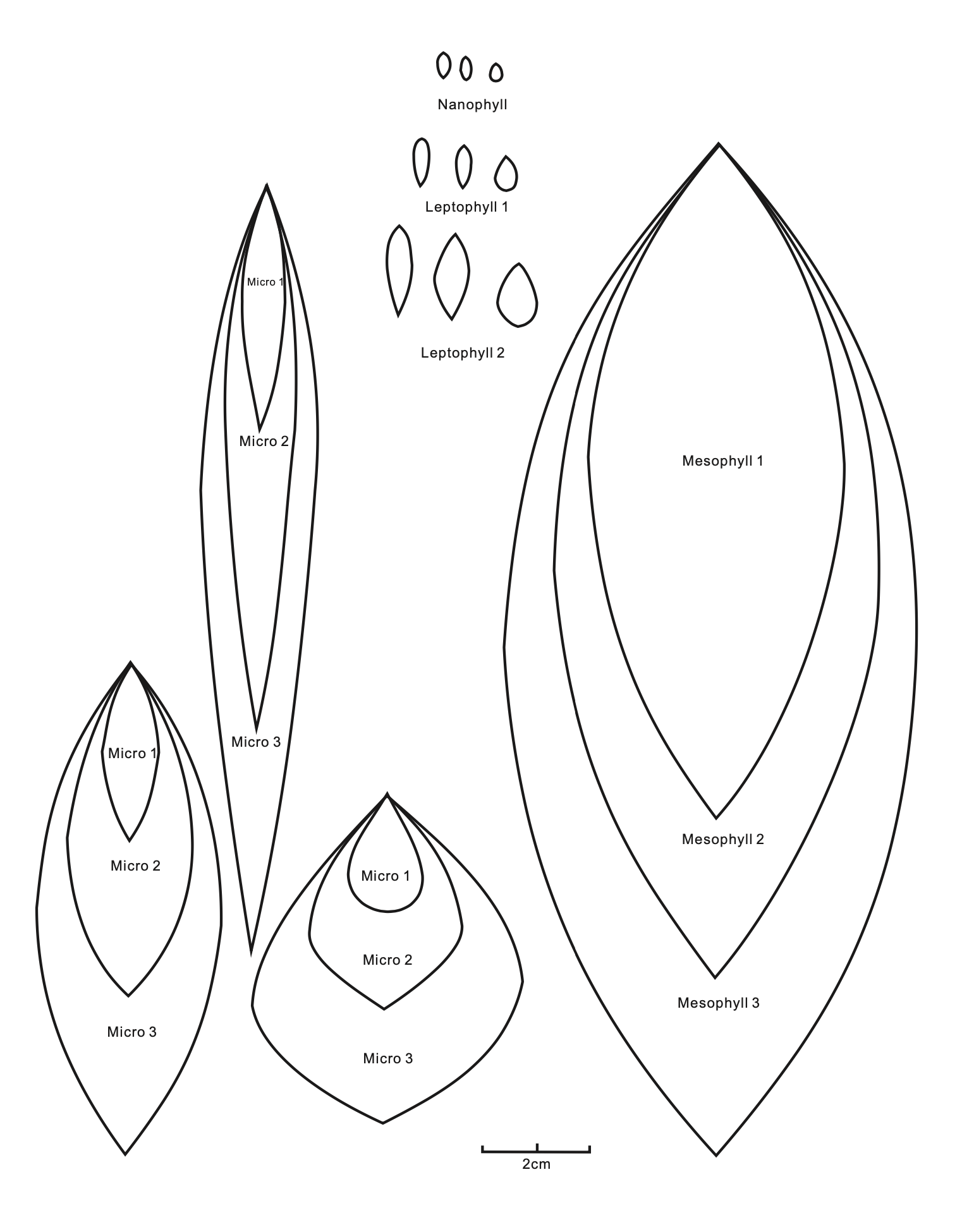


**Supplementary Figure 4: The boxplots showing the threshold of outliers flagged as unreliable measurements.**

Hard trait:


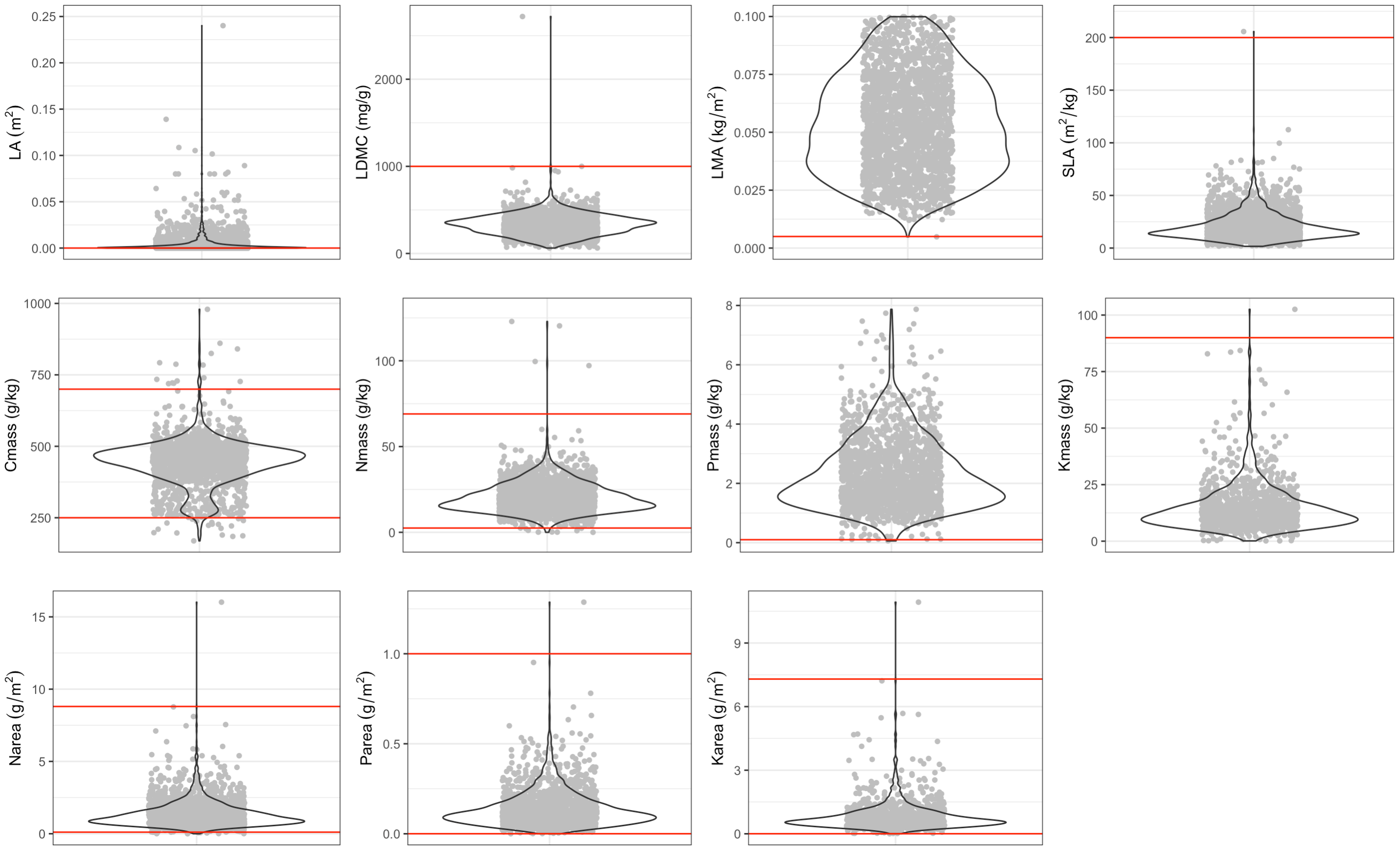


Photo trait:


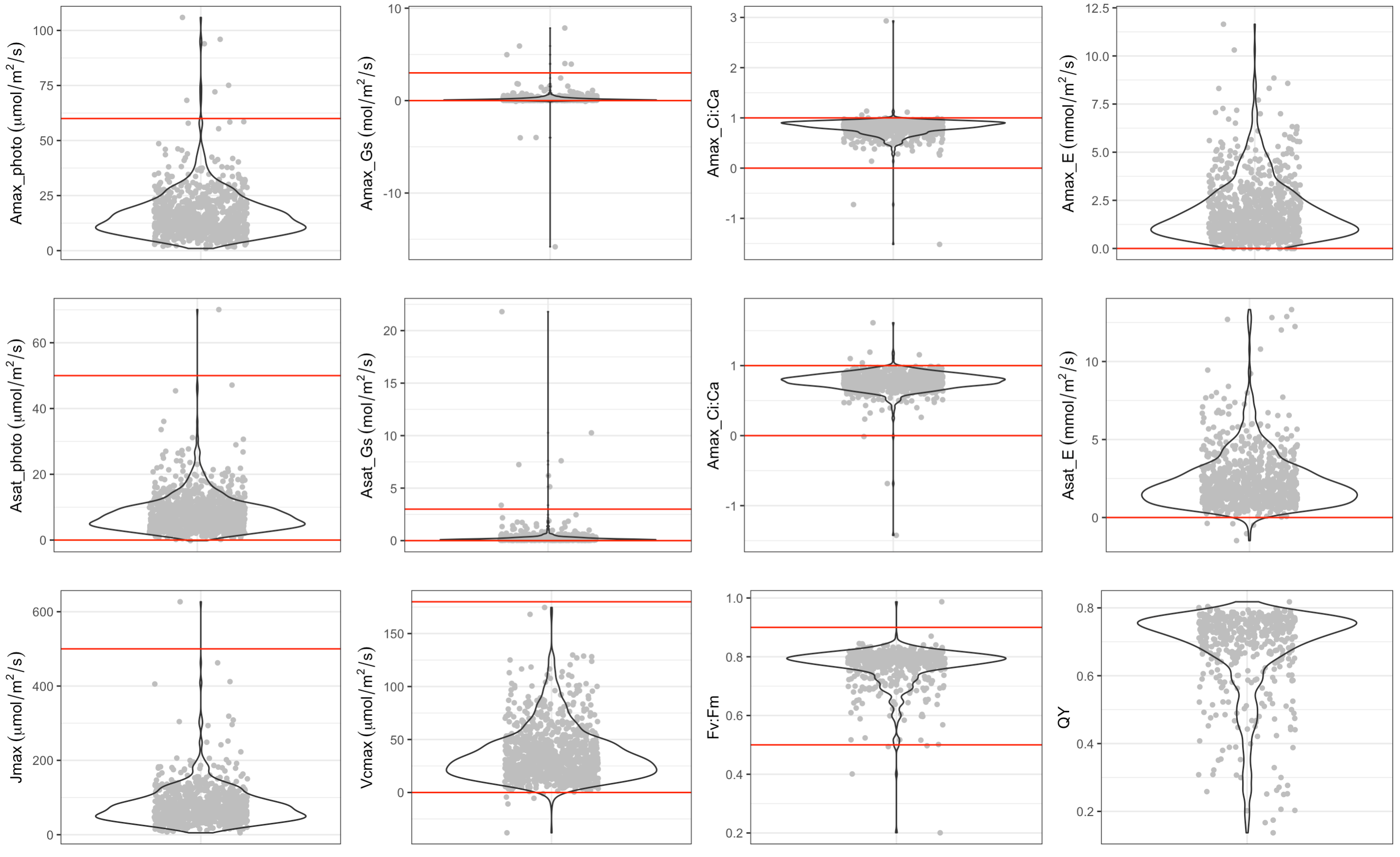

Supplement: Supplementary file 1 — Supplementary information [file 41597_2022_1884_MOESM1_ESM.docx]
